# Assessing de facto wastewater reuse and its implications for water quality in Yangtze Basin (2014–2021)

**DOI:** 10.1016/j.heliyon.2024.e40275

**Published:** 2024-11-08

**Authors:** Zhuomin Wang, Xueting Li, Shengchang Xiang

**Affiliations:** a Fujian Normal Univ, Coll Chem & Mat Sci, Fujian Prov Key Lab Polymer Mat, 32 Shangsan Rd, Fuzhou, 350007, Fujian, China; b South-Central Minzu University, School of Ethnology and Sociology, Hongshan District, Wuhan, Hubei, China

**Keywords:** Wastewater reuse, GIS modelling, Water quality assessment, Yangtze Basin

## Abstract

De facto wastewater reuse is the incidental presence of treated wastewater in a water supply source. Unplanned indirect wastewater reuse, which is also called de facto reuse, occurs when wastewater is discharged into surface water upstream of the intakes of suitable drinking water treatment plants. Although this discharged wastewater may increase the water quality risks for downstream water supplies, they contribute to the water supply source as an additional in-stream flow. Therefore, proper wastewater management is crucial to ensure access to safe water and address the challenges due to urbanization and population growth. There are detailed data on the infrastructure and river flows in some countries, but this study examined the use of a more limited dataset in other countries and specifically assessed the de facto reuse in the Yangtze River Basin in China. GIS modelling was used to calculate the streamflow of Yangtze River from the DEM, while macro water consumption data was used to estimate the wastewater discharges, and this methodology solved the problem that the outcome was limited by the lack of gauging stations at more locations and lack of precise geospatial location of drinking water intakes or wastewater discharges. Under an average-flow condition, Chongqing in the upper reaches had de facto reuses that increased from 0.57 % in 2014 to 0.60 % in 2021; Wuhan in the middle reaches had de facto reuses, which increased from 1.56 % in 2014 to 1.64 % in 2021; Shanghai, which is located in the estuary of the Yangtze River, increased its de facto reuse from 2.35 % in 2014 to 2.51 % in 2021. Under low-flow conditions, the de facto reuse of Chongqing, Wuhan, and Shanghai in 2021 was 1.7 %, 4.9 %, and 7.4 %, respectively. This relatively high level of de facto reuse is consistent with the estimation of unintended treated wastewater contributions to streams and growing risk of drinking water quality in the Yangtze River Basin. This research estimated de facto reuse in the Yangtze River Basin and showed the feasibility of using limited infrastructure datasets to assess de facto reuse and the modeling methods allow identification of river reaches most impacted by wastewater, and perhaps suggest prioritization for investments in additional wastewater treatment in reaches with high de facto reuse.

## 1 Introduction

Wastewater discharged into surface waters represents a potentially reliable water source, but it can convey chemical and microbial pollutants to cities and drinking water intakes located downstream. With rapid development of the economy and population, the number of new chemicals is growing from 20 million to 156 million between 2002 and 2019 in the Chemical Abstract Service Registry. The consequently increasing chemical contamination of freshwater systems becomes one of the key environmental problems facing humanity [1]. Modern wastewater treatment plants (WWTPs) discharge treated wastewater into rivers, which inevitably affects the water safety of cities downstream [2]. However, the unplanned reuse of water after treatment in WWTPs can be an underutilized but crucial aspect of sustainable water resource management. The National Academy of Engineering (NAE) in the United States published a lecture about wastewater reuse, which identified the need to quantify the extent of unplanned potable reuse as a top research priority for social and environmental studies. De facto wastewater reuse occurs when treated wastewater is discharged from WWTPs into surface waters upstream of potable drinking water treatment plant (DWTP) intakes [3]. De facto reuse is a prevalent and growing practice that can considerably contribute to the overall water source. It is not uncommon for a significant portion of the source water to originate from upstream wastewater. De facto wastewater reuse is the unplanned or incidental presence of treated wastewater in a water supply source. It is not uncommon for a substantial portion of the source water for DWTPs to be originally derived from upstream treated wastewater contributions to the surface water resource.

WWTP discharges introduce risks to downstream ecosystems and affect the health of people who use the river as a drinking water source. WWTP discharges are a significant source of micro pollutants in the environment [4], including endocrine disruptors, pharmaceuticals, personal care products, disinfection byproduct precursors, nutrients, and pathogens, which can pose ecological and human health risks [5,6]. GIS-based models have been developed in many regions to help managers assess potential risks from de facto reuse in basins [7,8].

The existing research on de facto reuse relies on large quantities of data on drinking water systems, including the locations and design flows of DWTPs and WWTPs and hydrological datasets [9,10]. These models work well and play an important role in drinking water management in regions with complete data. However, there are limited data on DWTPs, WWTPs, and hydrological dataset in many developing countries, and these regions lack information on the occurrence of de facto reuse. Wastewater pollution is an important bottleneck that restricts the global sustainable development. China is one of the largest contributors to wastewater discharges and eco-environmental damages in the world [11,12]. Can de facto reuse be analyzed under incomplete data conditions? Are the conclusions drawn from incomplete data different from those drawn from complete data? This paper uses the Yangtze Basin in China as a case study to analyze the de facto reuse and answer these questions.

**Fig. 1:**
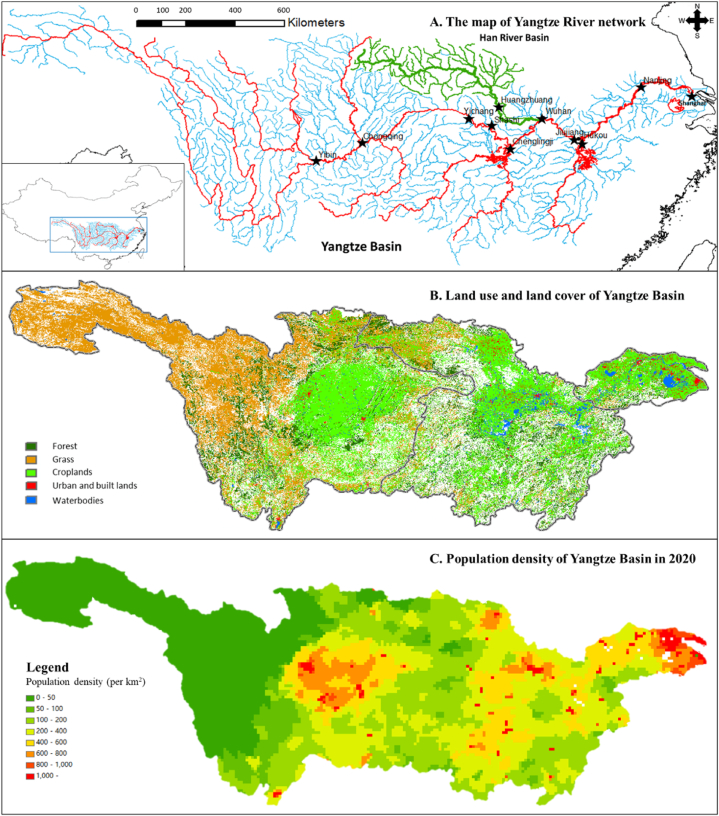
(A) Yangtze River basin and major cities; (B) LULC and boundary between upstream, midstream, and downstream of this basin; (C) population density map of this basin.

China has the largest population in the world and has been rapidly urbanizing and investing in water infrastructure, yet many regions are water stressed. Yangtze River showed in Fig. 1(A) is the third longest river in the world and the longest river in Asia, stretching over 6300 km from the Qinghai-Tibet Plateau to the East China Sea at Shanghai [13]. The Basin holds one-fifteenth of the world's population and one-third of China's water resources. Fig. 1(B) depicts the rivers, land use and land cover, the boundary between upstream, midstream, and downstream and population density of Yangtze Basin and highlights significant variations in population density and clear spatial relationships between upstream and downstream cities. The upper reach, except within the Sichuan basin, is characterized by higher elevations, lower average temperatures, mostly grass and forest lands, and low population density. Broad plains formed of alluvial deposits crisscross the middle reach of the basin. This area is known as China's major granary and is characterized by moderate temperatures and abundant rainfall that support large areas of no irrigated croplands. Population in major cities such as Wuhan and lands across the basin have seen more significant urbanization than the upper reaches, resulting in a loss of natural grass and forest lands. The Yangtze River delta in the lower reach includes the economic centers of Shanghai and Nanjing and is the most affluent region in China [14]. The provinces of China annually publish water resource bulletins that include macro data. Thus, the Yangtze Basin can be studied as an important case to examine de facto reuse from incomplete data. This research used data collected from these bulletins, including information on the water intake and wastewater discharge in the basin [15].

The purpose of this work is to assess the extent and new trends of de facto reuse in the Yangtze Basin from 2014 to 2021. The assessment consists of several components: (1) completing data through reasonable assumptions and the use of digital elevation models (DEMs); (2) using the ArcGIS framework to develop a model to determine the spatial relationships among the cities throughout the basin; (3) quantifying the amount of accumulated upstream wastewater of all cities in Yangtze Basin; (4) examining the wastewater percentages under average- and low-flow stream conditions in different years; and (5) analyzing the characteristic of de facto reuse in the Yangtze Basin. This renewed study about de facto reuse in the Yangtze Basin added data from 2014 to 2021, and it provides new insights into the water consumption and discharge trends in China. The reliability of the model to assess the de facto reuse was verified by comparison with previous research on 1998–2014 data. This work demonstrates the method to continuously improve the accuracy of the GIS model when more detailed data become available in the studied districts. Overall, this work provides a reliable and constantly updated model that can assist with drinking water management in this Basin.

## 2 Materials and methods

### 2.1 The method of calculation

To calculate the de facto reuse, conservative assumptions were made, including (1) WWTP discharge was equal to that of the plant design flow, (2) WWTP effluent had no in-stream loss, and (3) all water bodies were completely mixed. A mass balance was performed for the wastewater effluent at each DWTP intake point under the assumption that the WWTPs were the only input of wastewater and the DWTP was the sole uptake. Therefore, the wastewater percentage was calculated by dividing the total upstream discharge by the streamflow [16]. This study analyzed the de facto water reuse of several cities in the Yangtze Basin, and the concrete calculating method is as follows:

(1) $$\text{De}\;\text{facto}\;\text{reuse}=\frac{\sum_{i=1}^{N-1}D_{i}}{Q_{N}}$$

where N is the number of cities from upstream to downstream along the river;

Q_i_ is the streamflow of the river where city i intakes water;

and D_i_ (m^3^/s) is the amount of wastewater discharge from the wastewater treatment plant of city i.

When assessing de facto wastewater reuse, we calculated the wastewater percentage in the quantity of water intake under average- and low-flow stream conditions in different years. Based on Equation (1), the streamflow significantly contributes to the de facto reuse level over time. A high de facto reuse percentage poses high risks to the water quality and human health when the city has a low streamflow. The “low flow” of a stream indicates that the quantity of streamflow is comparatively low in prolonged periods, which is influenced by the local climate and characteristics of its drainage area. To compare the low-flow regimes of different basins, we used the 7Q10 statistic, which is commonly used to determine the permitted point-source pollutant levels in streams [17].

The 7-day-10-year (7Q10) low flow, as recommended by the United States Environmental Protection Agency, was used to evaluate the streamflow during periods of low flow. This approach involves fitting historical low-flow data to a specific probability density function and calculating the flow rate that has a 1/10 probability of being exceeded. The log Pearson Type III distribution was used for this purpose because it can accommodate many distributional shapes, which makes it a widely adopted method in stream flow frequency analysis [18].

The formula of the 7Q10 low flow using the log Pearson Type III method is as follows:

(2) 7Q10=exp(u+K∗S)

(3) $$K=\left(\frac{2}{g}\right)\left[\left(1+g*\frac{z}{6}-\frac{g^{2}}{36}\right)*3-1\right]$$

Where u is the mean of the logarithms (base e) of the historical annual low flows; K is the frequency factor for skewness coefficient g and a return period of 10; S is the standard deviation of the logarithms of the historical low flows; g is the skewness coefficient of the logarithms of the historical low flows; and z is a constant value of −1.123 that corresponds to a return period of 10.

### 2.2 The way of data acquisition

To achieve the goals of this research, data were collected from various sources. The primary large datasets consisted of data on the amount of water intake and wastewater discharge of each city in the Yangtze Basin, which were obtained from water resource bulletins published every year. Daily streamflow data of 11 hydrological stations showed in Fig. 1 (A) from 1954 to 2021 were collected from the Yangtze River Water Resources Committee Website (www.cjw.gov.cn), and the other streamflow data would be calculated using ArcGIS's hydrological analysis tools.

Eleven representative cities were selected for this research based on their varied geographic location along the Yangtze River. The Yangtze River serves as a crucial source of potable drinking water for several populous urban areas along its course. Chongqing, Wuhan, and Nanjing are significant urban centers in the upper, middle, and lower reaches of the Yangtze Basin, respectively. Shanghai, which is positioned at the estuary of the Yangtze River, is the largest commercial city and economic hub in China. Chenglingji, Hukou, and Huangzhuang have hydrological stations in the Dongting Lake, Poyang Lake, and Han River, respectively. The GIS revealed that these cities discharge treated wastewater into and intake drinking water from the Yangtze River.

**Fig. 2:**
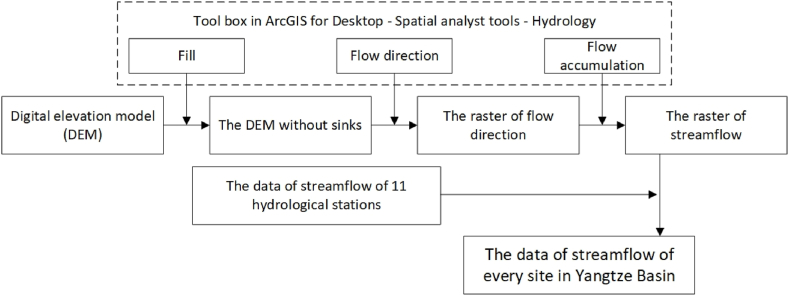
Flowchart of the use of the DEM and hydrological analyst tool to obtain streamflow data.

This research used the hydrological analyst tool in ArcGIS and DEM to obtain the streamflow data of regions without hydrological stations in Yangtze Basin. Fig. 2 shows the flowchart of using the DEM and hydrological analyst tool to obtain streamflow data. We used the module “Hydrology” to calculate the streamflow of Yangtze River from the DEM. Previous studies have proven that the application of slope, land use, soil, climate and other data influence the runoff of river systems. When DEM was used in hydrological analyst, there were some problems like the discontinuous dislocation of empty area and poor connectivity of the water system. These were mainly due to the complexity of underlying surface conditions and the uneven distribution of hydro-meteorological elements. The accuracy of the model was greatly improved when the catchment area threshold was checked and calculated repeatedly, the threshold for confluence accumulation was set to 2000 in this case, and a sub-basin fusion processing on discontinuously dislocated areas was performed [19]. The first step was using the tool box “Fill” of Hydrology in the Spatial analyst tools to generate the DEM without sinks because the DEM is a relatively smooth terrain surface model, and a small part of the DEM has a depressed area. Next, the tool box “Flow Direction” was used to generate the raster of flow direction, and the raster of streamflow was calculated using the toolbox “Flow Accumulation.” The ratio for streamflow data of each site was assumed to slightly change over time, so we obtain the streamflow data of every site in the Yangtze Basin by comparing different values of the streamflow raster in different sites.

We obtained base layers for topography, stream networks, and city boundaries from the China Geological Survey Website (www.cgs.gov.cn). The model was built using ArcGIS 10 as the framework and data analysis platform that provided spatial context for the locations of cities in the Yangtze Basin. We incorporated data from various sources into the GIS model, which will help us assess the de facto reuse in the Yangtze Basin.

## 3 Results and discussion

### 3.1 Population and wastewater characteristic

In the 21st century, China experienced great changes in almost every field including population growth and economic development. China had 7 ‰ population growth in 2001 and 6.7 ‰ in 2014. The population growth number was only 3.8 ‰, 3.3 ‰, 1.4 ‰, and 0.3 ‰ in 2018, 2019, 2020, and 2021, respectively. In 2022, China's population decreased for the first time in 61 years, with a decrease of 850,000 from 2021. Simultaneously, the economic growth also had a downward trend: the gross domestic product (GDP) growth was 8.3 %, 7.4 %, 6.7 %, 6 %, 2.2 %, 8.1 %, and 3 % in 2001, 2014, 2018, 2019, 2020, 2021, and 2022, respectively (China had economic fluctuations because of the COVID-19 epidemic from 2020 to 2022). The information on China population and economic growth was collected from the State Information Center of China (www.ceidata.cei.cn).

The Yangtze River, which flows from west to east, is a crucial waterway, and the Yangtze Basin is an important region in China [20]. In the upper reaches, the Yangtze River flows from the plateau to the plain, which results in steep gradients, plentiful water quantities, and high flow velocities. In the middle reaches, the gradient of the Yangtze River decreases, and the course meanders, which leads to a lower flow velocity and a broader river surface. Thus, this region has the widest area, receives the most precipitation, and has a dramatic increase in water volume. In the lower reaches, the Yangtze River flows more slowly, and the basin has a flat terrain and a dense water network; this region has the densest population and the most developed economy.

**Fig. 3:**
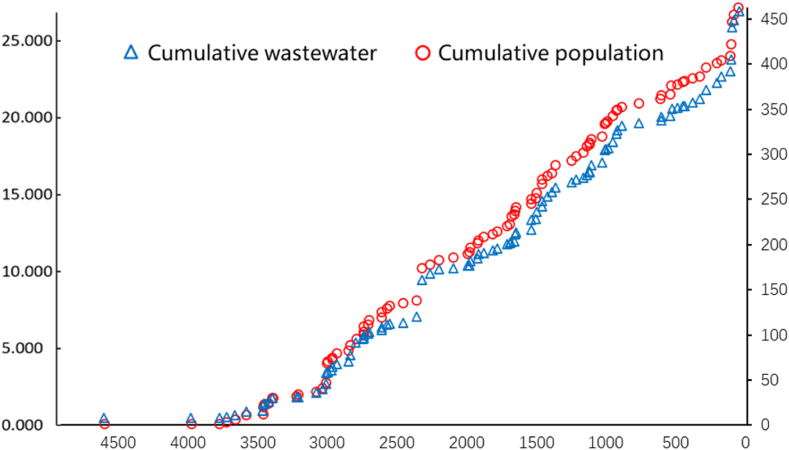
Cumulated distribution of wastewater and population of 97 cities in the Yangtze Basin in 2021.

**Fig. 4:**
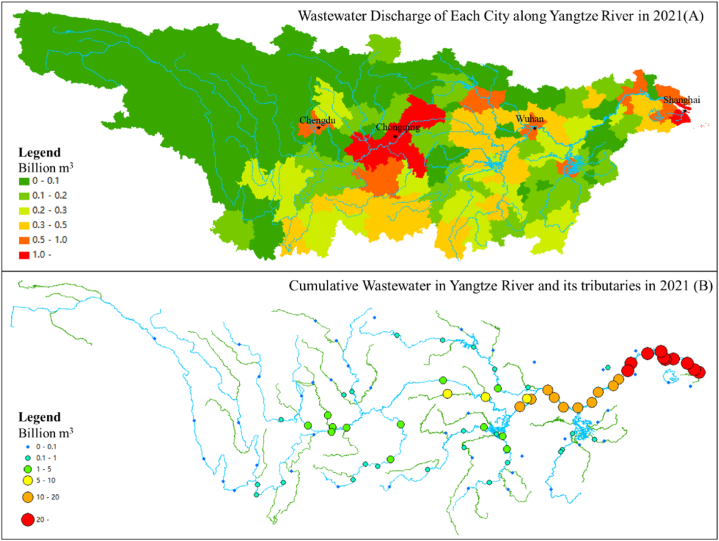
Wastewater discharge and cumulative wastewater of each city in the Yangtze Basin in 2021.

Fig. 3 shows the cumulated wastewater discharge and cumulated population distribution of 97 cities in the Yangtze Basin in 2021. The x-axis of Fig. 4 indicates the distance between each city and the estuary into the East China Sea along the river, which briefly shows the spatial relationship between each city. The shortest path method in ArcGIS along the river was calculated for these distances.

Fig. 3 highlighted how a relatively clean river can be contaminated by untreated wastewater released from each city in this basin. In particular, the lower Yangtze River (LYR) provides 80 % and 100 % of the drinking water to the densely populated and economically developed Jiangsu Province and Shanghai Municipality, respectively, supporting almost 88 million permanent inhabitants. In the last two decades, the discharge of pollutants into the Yangtze River has increased by 41 % in response to intensive anthropogenic perturbations and resulted in a dramatic deterioration of water quality [21]. In this basin, A range of organic contaminants, including pharmaceuticals (APIs), personal care products (PCPs), hormones (EACs) and industrial chemicals, were detected along the river flowing through the urban centre in middle and lower reaches of the Yangtze River. Although there was no definitive point source for these contaminants, their concentrations increased along with the extent of urbanization and subsequent population density. Indeed, the concentrations of many of these contaminants remained consistent, or increased, downstream of the geographical limits of the urban centre, indicating very little attenuation occurred in the Yangtze River [22]. This result was consistent with research conducted in other regions [23,24].

In 2021, the upstream region included 39 cities in total, which constituted approximately 37 % of the total population and accounted for 35 % of the wastewater. The midstream region consisted of 41 cities, which constituted approximately 40 % of the population and accounted for 38 % of the wastewater. The downstream region consisted of 17 cities, which constituted approximately 23 % of the population and accounted for 27 % of the wastewater. Comparatively, in 2010, the proportions of population and wastewater distribution were slightly different, where the upstream, midstream, and downstream regions accounted for 40 %, 37 %, and 23 % of the population and 37 %, 35 %, and 28 % of the wastewater, respectively.

The differences between the percentages of population and wastewater discharge are caused by various geographical, population, and economic characteristics in the upper, middle, and lower reaches of the Yangtze Basin. The upstream region has a large area, a high flow velocity, numerous canyons, a rapid water flow, abundant hydropower resources, a small-density population, and a small quantity of wastewater discharge. The upstream region also has few human activities and discharges a small amount of wastewater, so the cumulated distribution of wastewater and population slowly increased along the river. The midstream region has a curved river, abundant water resources, fertile land, a high-density population, and a highly developed agricultural system with warm climate. The midstream region has human activities and discharges a large amount of wastewater, so the cumulated distribution of wastewater and population rapidly increased. The downstream region has a flat terrain, a wide river surface, a slow streamflow rate, the most prosperous economy, the highest population density, and a high volume of wastewater. The downstream region has extensive human activities and discharges a huge amount of wastewater, so the cumulative wastewater and population also quickly increased here.

During the period from 2010 to 2021, the portion of upstream region population and wastewater discharge in the Yangtze Region decreased due to human migration and the ecological protection in the upper reaches of the Yangtze River. In the midstream region, the percentage of population and wastewater discharge increased by 3 % in this period due to faster economic growth. The downstream region had an economic development level and an environmental protection level similar to those of the developed region, so the wastewater discharge percentage decreased by 1 %, whereas the population percentage remained basically unchanged in the Yangtze Basin.

### 3.2 Analysis of the de facto reuse in average flow condition

Fig. 4(A) shows the amount of wastewater discharge of each city in the Yangtze Basin. When the wastewater was discharged into the Yangtze River and its tributaries, the wastewater cumulated along the river. Fig. 4(B) shows the cumulated wastewater in the Yangtze River and its tributaries, which was obtained by identifying the water intake and wastewater discharge locations of every city and calculating the quantity of cumulative wastewater in the Yangtze Basin.

The upstream region has the geographical features of a high altitude, low temperature, and abundant gorges, so most cities have small populations and poor economic development. Thus, the quantity of wastewater discharge is small, and wastewater slowly cumulates along the river. Chongqing with 32.1 million residents and Chengdu with 21.2 million residents significantly contribute to the cumulated wastewater in the upper reaches of the Yangtze Basin. The cities in the midstream region had larger populations, more economic development, and more water consumption than most cities in the upstream region. Thus, the quantity of wastewater discharge and cumulative wastewater rapidly increased in the midstream region, especially in three cities with flourishing heavy industry: Xiangyang, Wuhan, and Nanchang. The downstream region of the Yangtze Basin, which is also named the Yangtze River Delta Region, is one of the prosperous regions in China. Here, the cities have large populations, well-developed industries, and abundant water consumption, so the quantity of wastewater discharge in each city is huge. When the Yangtze River flows to the estuary, it cumulates all of the wastewater in the basin, so the quantity of cumulated wastewater is enormous in the downstream region.

Based on Figs. 1 and 11 locations across the Yangtze Basin were selected for the watershed-scale case studies. Chongqing, Wuhan, and Nanjing are major cities in the upper, middle, and lower reaches of this basin, respectively; Shanghai is the largest city in China and is located at the estuary of the river. Yibin, Yichang, Shashi, and Jiujiang are located in the main stream of the Yangtze River and have rich hydrological data. Chenglingji, Hukou, and Huangzhuang are regions with hydrological stations in the Dongting Lake, Poyang Lake, and Han River, respectively. The online survey on DWTPs and WWTPs shows that in most of these cities, DWTPs intake drinking water from and WWTPs discharge wastewater into the Yangtze River and its tributaries. Annual data on the water intake, water consumption, and wastewater discharge were used because data are available in annually published water resource bulletins. Specific years were selected for the data analysis due to desirable variations: 2014, 2018, and 2021. The values in these 3 years were used to analyze the de facto reuse. This study used Eq. (1) to calculate the potential de facto reuse at each location. The cumulative upstream wastewater data were collected from the water resource bulletin. The streamflow data were obtained from hydrologic gauging stations or estimated streamflow using the computing method illustrated in Fig. 2.

First, the change in cumulative upstream wastewater flow was quantified to assess the de facto reuse changes from 2014 to 2021. During this period, the wastewater effluent contribution to the Yangtze River increased by 13.1 % (from 22.30 billion m^3^ to 25.23 billion m^3^). The wastewater effluent contribution to the Han River and Dongting Lake increased by 13.3 % (from 0.98 billion m^3^ to 1.11 billion m^3^) and 26.6 % (from 3.16 billion m^3^ to 4.00 billion m^3^), respectively, whereas the wastewater effluent contribution to the Poyang Lake decreased by 13.0 % (from 1.92 billion m^3^ to 1.67 billion m^3^). All sites except Hukou (Poyang Lake) experienced increased wastewater contributions from upstream WWTPs in 2021. Specifically, in the upper reach, Chongqing observed an 11.3 % increase (0.22 billion m^3^) in wastewater flow. In the middle reach, Wuhan's wastewater flow increased by 13.6 % (1.53 billion m^3^). In the lower reach, Nanjing experienced an 11.7 % increase (2.18 billion m^3^). The largest change occurred in Shanghai, i.e., the largest city in the Yangtze River Basin near the river estuary, with a 13.1 % increase (2.93 billion m^3^) during this period.

The previous research showed the overall concentrations of volatile methyl siloxanes varied from upper to the lower reaches and the concentrations followed an order of lower > upper > middle reaches [25]. Volatile methyl siloxanes in surface water was well positively correlated with this in the wastewater, and the correlations also showed the significant contributions of wastewater from industrial applications to the water qualities, so this research was consistent with the increasing trends of cumulative wastewater showed in this section.

**Fig. 5:**
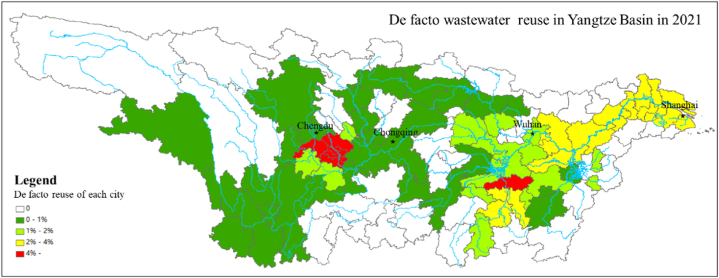
De facto reuse under average streamflow of each city in the Yangtze Basin in 2021.

Fig. 5 illustrates the potential de facto reuse of each city in the Yangtze Basin in 2021.The cities in the bottom of the Yangtze Basin had high de facto reuse percentage because they had the highest cumulative wastewater flow. The cities along tributaries with high populations and low-flow streams also had high de facto reuse percentages, which implies that these cities had a high water quality risk. Additionally, Fig. 5 shows that the cities in the upper reach of the Yangtze River or its tributaries had a low percentage of wastewater reuse in water intake, which indicates a low water quality risk in these cities. The cities in the lower reach, especially those with large populations upstream along the river, had a higher risk of water contamination.

The de facto reuse was estimated using the annual average streamflow conditions, assuming that DWTPs intake drinking water from the Yangtze River in these sites. Table 1 shows that the amount of de facto reuse increased at nearly all sites except Huangzhuang (Han River) from 2014 to 2021.The de facto reuse in Chongqing, Wuhan, and Nanjing increased from 0.57 % to 0.60 %, from 1.56 % to 1.64 %, and from 2.04 % to 2.15 %, respectively, which represent the characteristic of the changes in de facto reuse in the upper, middle, and lower reaches in the Yangtze Basin. The de facto reuse in the Dongting Lake and Poyang Lake increased from 1.13 % to 1.50 % and from 1.22 % to 1.23 %, respectively. However, the de facto reuse in the Han River decreased from 4.74 % to 1.52 % because the streamflow in this river (Huangzhuang site) dramatically increased from 21 billion m^3^ to 74 billion m^3^, although the cumulative upstream wastewater flow increased by 13.3 % from 0.98 billion m^3^ to 1.11 billion m^3^.

**Table 1:** Cumulative upstream wastewater flow, streamflow, and de facto reuse under average streamflow of 11 sites (billion m^3^/year).

City	Cumulative upstream wastewater flow (billion m^3^/year)			Streamflow (billion m^3^/year)			De facto reuse under average streamflow		
	2014	2018	2021	2014	2018	2021	2014	2018	2021
Yibin	1.30	1.43	1.46	128	164	123	1.01 %	0.87 %	1.19 %
Chongqing	1.95	2.17	2.17	344	387	361	0.57 %	0.56 %	0.60 %
Yichang	7.10	7.09	7.83	449	474	472	1.58 %	1.50 %	1.66 %
Shashi	7.36	7.34	8.40	417	433	435	1.76 %	1.70 %	1.93 %
Wuhan	11.30	11.30	12.84	723	670	783	1.56 %	1.69 %	1.64 %
Jiujiang	14.72	14.79	17.13	733	679	794	2.01 %	2.18 %	2.16 %
Nanjing	18.57	18.40	20.75	909	803	965	2.04 %	2.29 %	2.15 %
Shanghai	22.30	22.58	25.23	948	837	1006	2.35 %	2.70 %	2.51 %
Huangzhuang (Han River)	0.98	0.90	1.11	21	38	74	4.74 %	2.38 %	1.52 %
Chenglingji (Dongting Lake)	3.16	3.08	4.00	279	199	267	1.13 %	1.55 %	1.50 %
Hukou (Poyang Lake)	1.92	1.86	1.67	157	104	136	1.22 %	1.80 %	1.23 %

The Yangtze River Delta city cluster is the most prosperous and densely populated area in China, however, the long-term excessive pursuit of high-speed economic growth has led to a series of problems such as disparity in regional development, vicious regional competition, over-consumption of resources, and serious deterioration of regional ecosystems. In this region, the total growth of regional GDP has long been the main variable in the performance assessment, and local governments are engaged in economic growth strategies in order to increase the proportion of regional GDP, resulting in the rising environmental and public health risk.

Compared to other study cases about de facto reuse [3,16,26], it could be found the proportion of de facto reuse in Yangtze Basin was not extremely high, especially in the city located in main stream of Yangtze River. The World Health Organization has employed a guideline stating that wastewater contributions to a drinking water source should be less than 10 % to avoid any chemical hazard. Using this guideline as a rubric, 1 of the 97 cities in this basin in 2021 update exceed this limit under average long-term streamflow conditions. However, the trend of de facto reuse continuous growth demands greater attention in this basin.

### 3.3 Analysis of the de facto reuse in low flow condition

**Fig. 6:**
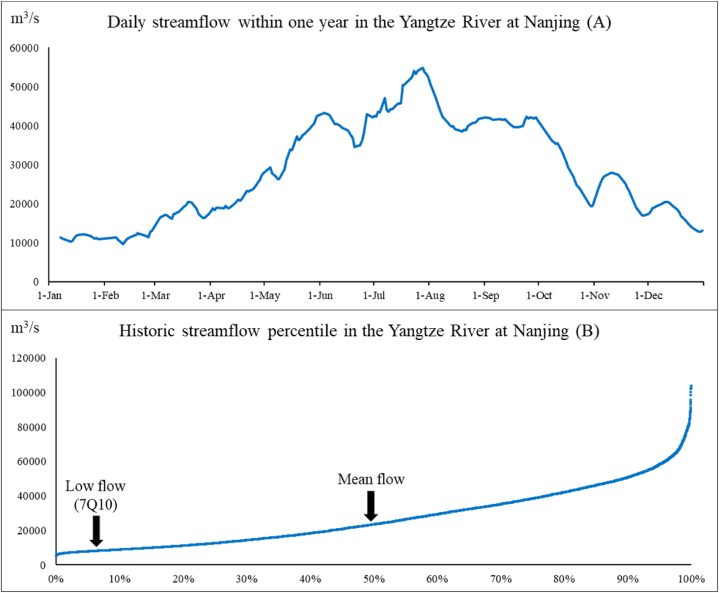
(A) Daily streamflow and (B) historic streamflow percentile of the Yangtze River at Nanjing.

The analysis presented in Section 3.2 reveals that streamflows significantly contribute to this process. To explore this relationship, we conducted a study with Nanjing as an example. The gauging streamflow data from Datong hydrological station near Nanjing were used to plot the wastewater effluent percentages against the full range of flow percentiles. Fig. 6 (A) uses the streamflow data of Nanjing in 2014 to show the changes in daily streamflow within 1 year in the Yangtze River; the minimum and maximum streamflow volumes in this year were 9730 m^3^/s (11-Feb) and 54800 m^3^/s (28-Jul), respectively. Fig. 6 (B) shows the frequency distribution of streamflows (m^3^/s) at this station and indicates that the streamflows greatly varied for different percentiles.

It can be assumed wastewater discharge from each city only slightly changed over time because the production and water consumption of each city were stable, but the streamflow from each site widely varied over time. Thus, the streamflow significantly contributes to the de facto reuse level over time. A high de facto reuse percentage poses high risks to the water quality and human health when the city has a low streamflow. Since a high de facto reuse percentage is caused by a low streamflow, the 7Q10 streamflow occurrence was considered as a near “worst-case” scenario for the minimum streamflow and the highest de facto reuse in different cities.

In the upper reaches, Yibin and Chongqing experienced increases in de facto reuse from 2.8 % in 2014 to 3.1 % in 2021 and from 1.6 % in 2014 to 1.7 % in 2021, respectively. In the middle reaches, Yichang, Shashi, and Wuhan saw increases in de facto reuse from 4.4 % in 2014 to 4.8 % in 2021, from 4.9 % in 2014 to 5.6 % in 2021, and from 4.3 % in 2014 to 4.9 % in 2021, respectively. In the lower reaches, Jiujiang increased its de facto reuse from 5.6 % in 2014 to 6.5 % in 2021; Nanjing increased its de facto reuse from 5.6 % in 2014 to 6.3 % in 2021; Shanghai in the estuary had the highest de facto reuse level among mainstream cities, which increased from 6.5 % in 2014 to 7.4 % in 2021. Chenglingji along the Dongting Lake increased its de facto reuse level from 3.1 % in 2014 to 4.0 % in 2021, but Hukou along the Poyang Lake decreased its de facto reuse level from 3.4 % to 2.9 %. Huangzhuang, which is situated on a major tributary (Han River) of the Yangtze River, had the highest de facto reuse in these sites, which increased from 13.1 % in 2014 to 14.8 % in 2021.

Taking into temporal streamflow variations consideration, nearly one fourth of the cities in Yangtze Basin would exceed 10 % wastewater contributions to drinking water source in low flow condition, in other words, these cities would encounter chemical hazard in drinking water at some point during the whole year. When the streamflow at the water intake of a certain city decrease to a certain extent, it is suggested this city should intake drinking water from reservoir instead of Yangtze River to avoid any chemical hazard. The trends found in this section indicate the need for a similar analysis to be performed for the remaining DWTPs across the basin.

## 4 Summary and conclusion

This research used a limited infrastructure dataset to build a GIS model to estimate the quantity of de facto wastewater reuse in the Yangtze Basin during 2014–2021. The key findings are summarized below.

1. In this research, the object of study lacks information on the de facto reuse occurrence, e.g., the hydrological dataset was limited, and there were imprecise locational and design data about the DWTPs and WWTPs. Macro water consumption data were used to complete the data of a drinking water system; a DEM and a geographical analyst tool were used to complete the hydrological data. De facto reuse was studied from incomplete data with reasonable assumptions. The model of de facto water reuse assessment from a limited dataset was credible, and it can be beneficial for drinking water management, especially in developing countries.
2. The quantities of municipal upstream wastewater flow and streamflow contribute to the de facto reuse. Under an average-flow condition, Chongqing in the upper reaches had de facto reuses that increased from 0.57 % in 2014 to 0.60 % in 2021; Wuhan in the middle reaches had de facto reuses, which increased from 1.56 % in 2014 to 1.64 % in 2021; Shanghai, which is located in the estuary of the Yangtze River, increased its de facto reuse from 2.35 % in 2014 to 2.51 % in 2021. Under low-flow conditions, the de facto reuse of Chongqing, Wuhan, and Shanghai in 2021 was 1.7 %, 4.9 %, and 7.4 %, respectively. This relatively high level of de facto reuse i increased the risk of quality safety management on drinking water in the Yangtze River Basin [27] and estimation of unintended treated wastewater contributions to streams in the Yangtze River Basin [28].
3. Cities in the lower reaches are more likely to withdraw a large amount of cumulative municipal wastewater. The de facto reuse level is affected by the cumulative upstream municipal wastewater, which has a close relationship with the upstream population density. The regions with high population density and low streamflow have high de facto reuse levels.
4. From 1998 to 2014, the de facto reuse percentages under low-flow conditions in the upper reaches (Chongqing), middle reaches (Wuhan), and lower reaches (Shanghai) increased by nearly 0.15 %, 0.13 %, and 0.35 % per year, respectively. The de facto reuse percentage and water risk rapidly increased during this time. From 2014 to 2021, China underwent a transition period of economic and social development and emphasized the increase in water efficiency, so the de facto reuse percentage under low-flow conditions in the upper reaches, middle reaches, and lower reaches increased by nearly 0.06 %, 0.07 %, and 0.13 % per year, respectively. The trend of rapidly increasing de facto reuse has changed. The water consumption of cities in the Yangtze Basin slightly improved with the increase in water efficiency, and the wastewater discharge slightly improved because the number of WWTPs continuously increased with the significant focus on water environment regulation and the financial support. During this period, the rate of population and economic growth also steadily declined, and the rate of cumulative municipal wastewater growth simultaneously decreased.

This work examined the potential risks associated with wastewater discharge and human health. Previous studies suggested that wastewater contributions to drinking water sources should not exceed 10 % to avoid chemical hazards [29]. Using this guideline, only few cities in the Yangtze Basin exceeded this limit under average long-term streamflow conditions. However, approximately one-fourth of cities in the middle and lower reaches of the basin exceeded this limit under low-flow conditions. These findings help managers assess potential risks from de facto reuse, help each city decide the water intake location at certain time, and highlight the need for further analysis of DWTPs in this basin.

The constructed model to assess de facto reuse in this research can identify river segments that are particularly affected by wastewater. By targeting reaches with high de facto reuse levels, investments in additional wastewater treatment can be strategically allocated to maximize the benefits of improving the water quality in the Yangtze River and its tributaries. The findings of this study can be used to determine suitable sites for enhanced collection of water quality data to more comprehensively characterize and assess the river reaches that are most impacted by wastewater (i.e., those with the highest levels of de facto reuse). Subsequent investigations into these regions can contribute to a better understanding of the impacts of water quality on human ecosystems.

This research proposed a model to assess the de facto reuse in the Yangtze Basin with limited datasets and some limitations. We made some simplifications in the geographic information system; for example, we assumed that all water intake and sewage were located at the main stream of the Yangtze River that flowed through each city, which is acceptable on the basin scale but inaccurate in some cities. In future studies, the estimation of the de facto reuse and assessment of the intake water risk will be more accurate with increasing attention being paid to wastewater discharge and water quality problems in China.

## CRediT authorship contribution statement

**Zhuomin Wang:** Writing – original draft, Visualization, Validation, Software, Resources, Methodology. **Xueting Li:** Writing – review & editing, Funding acquisition, Data curation. **Shengchang Xiang:** Writing – review & editing, Supervision, Investigation, Data curation.

## Data availability statement

Data included in article is referenced in the article.

## Declaration of competing interest

The authors declare the following financial interests/personal relationships which may be considered as potential competing interests:Shengchang Xiang reports financial support was provided by 10.13039/501100001809National Natural Science Foundation of China. Xueting Li reports financial support was provided by Fundamental Research Funds for the Central Universities. If there are other authors, they declare that they have no known competing financial interests or personal relationships that could have appeared to influence the work reported in this paper.
